# *Ab initio* crystal structures and relative phase stabilities for the aleksite series, Pb_*n*_Bi_4_Te_4_S_*n*+2_

**DOI:** 10.1107/S2052520623008776

**Published:** 2023-11-01

**Authors:** Jie Yao, Cristiana L. Ciobanu, Nigel J. Cook, Kathy Ehrig

**Affiliations:** a School of Chemical Engineering, The University of Adelaide, North Terrace, Adelaide, South Australia 5005, Australia; b BHP Olympic Dam, 10 Franklin Street, Adelaide, S.A. 5000, Australia; Czech Academy of Sciences, Czechia

**Keywords:** aleksite series, mixed layer compounds, crystal structure, density functional theory, electron diffraction

## Abstract

Density functional theory methods are applied to crystal structures and stabilities of phases from the aleksite homologous series, Pb_*n*_Bi_4_Te_4_S_*n*+2_ (*n* = homologue number). The seven phases investigated correspond to *n* = 0 (tetradymite), 2 (aleksite-21R and -42R), 4 (saddlebackite-9H and -18H), 6 (unnamed Pb_6_Bi_4_Te_4_S_8_), 8 (unnamed Pb_8_Bi_4_Te_4_S_10_), 10 (hitachiite) and 12 (unnamed Pb_12_Bi_4_Te_4_S_14_). These seven phases correspond to nine single- or double-module structures, each comprising an odd number of atom layers, 5, 7, (5.9), 9, (7.11), 11, 13, 15 and 17, expressed by the formula: *S*(*M*_*p*_ *X*_*p*+1_)·*L*(*M*_*p*+1_ *X*_*p*+2_), where *M* = Pb, Bi and *X* = Te, S, *p* ≥ 2, and *S* and *L* = number of short and long modules, respectively. Relaxed structures show *a* and *c* values within 1.5% of experimental data; *a* and the interlayer distance *d*_sub_ decrease with increasing PbS content. Variable Pb—S bond lengths contrast with constant Pb—S bond lengths in galena. All phases are *n*-fold superstructures of a rhombohedral subcell with *c*/3 = *d*_sub_*. Electron diffraction patterns show two brightest reflections at the centre of *d*_sub_*, described by the modulation vector **q**_*F*_ = (*i*/*N*) · *d*_sub_*, *i* = *S* + *L*. A second modulation vector, **q** = γ · *c*_sub_*, shows a decrease in γ, from 1.8 to 1.588, across the *n* = 0 to *n* = 12 interval. The linear relationship between γ and *d*_sub_ allows the prediction of any theoretical phases beyond the studied compositional range. The upper PbS-rich limit of the series is postulated as *n* = 398 (Pb_398_Bi_4_Te_4_S_400_), a phase with *d*_sub_ (1.726 Å) identical to that of trigonal PbS within experimental error. The aleksite series is a prime example of mixed layer compounds built with accretional homology principles.

## Abstract 2

DFT is used to obtain structural information for seven members of the aleksite homologous series. Relationships between modulation vectors and the *d* subcell can be mathematically defined, allowing the prediction of crystal parameters for any member of the series, a valuable finding for mineral systematics and classification, and for an expanded understanding of this and other mixed-layer series.

## 1. Introduction

Several named minerals and a number of unnamed Bi–Pb–telluro­sulfide phases were initially grouped together within a homologous series with the common formula Pb_*n*_Bi_4_Te_4_S_*n*+2_, where *n* is homologue number (Cook *et al.*, 2007*a* ▸). Later, the series was termed the aleksite series after the first named mineral, aleksite (Pb_2_Bi_4_Te_4_S_4_), with the generalized formula revised to Pb_(*n*−1)_Bi_2_ *X*_*n*+2_ (*n* = homologue number, *X* = chalcogen) (Moëlo *et al.*, 2008 ▸). Cook *et al.* (2007*a* ▸) postulated the existence of a hierarchical series of Pb–Bi–telluro­sulfides that can be expanded from the archetypal five-atom tetradymite unit to larger seven-, nine-, 11-atom units, whereas Moëlo *et al.* (2008 ▸) considered the tetradymite (Bi_2_Te_2_S) archetype as a link to layered sulfosalts. A second large group of minerals and unnamed compounds, Bi_*x*_ *X*_*y*_ (*X* = chalcogen), is also derived from the same archetype, constituting the tetradymite homologous series (Cook *et al.*, 2007*b* ▸).

Ciobanu *et al.* (2009 ▸) affirmed that the two homologous series derived from the tetradymite archetype share structural building principles in agreement with the formulae: (i) chalcogen-rich [S(*M*_*p*_ *X*_*p*+1_)·*L*(*M*_*p*+1_ *X*_*p*+2_]; *p* ≥ 2)] for the aleksite series, and (ii) bis­muth-rich [*S*′(Bi_2*k*_ *X*_3_)·*L*′[Bi_2(*k*+1)_ *X*_3_]) for the tetradymite series. Investigation of compounds from the tetradymite series in the compositional range Bi_2_ *X*_3_–Bi_8_ *X*_3_ using transmission electron microscopy (TEM) (Ciobanu *et al.*, 2009 ▸) confirmed that these are all *n*-fold (*N* = layers in the stacking sequence) superstructures of a rhombohedral subcell with *c*/3 = *d*_0_ ∼ 2 Å. Electron diffraction patterns show two brightest reflections in the centre of *d*_0_ and are described by two modulations vectors: **q** = 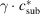 (**q** ∼ homoatomic interval) and **q**_*F*_ = 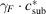 ; **q**_*F*_ = (*i*/*N*)*d*_0_* = 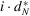 , *i* = *S*′ + *L*′.

The same basis for crystal structural modularity attributable to other mixed layer compounds (Amelinckx *et al.*, 1989 ▸; Frangis *et al.*, 1990 ▸) should extend to the aleksite series since their building modules follow the same accretional principle with the tetradymite compounds. This was demonstrated in a high-angle annular dark-field scanning TEM (HAADF STEM) study of unnamed PbBi_4_Te_4_S_3_ (Cook *et al.*, 2019 ▸). Based on the polytypism observed, Cook *et al.* (2019 ▸) showed that for a single homologue, *n* = 1 in this case, the structure could consist of combinations of multiple *S* and *L*_*m*_ modules, at constant *p* = 2.

*Ab initio* calculation of structures from the tetradymite series within the compositional interval Bi_2_Te_3_–Bi_8_Te_3_ has confirmed crystal structural modularity using the accretional formalism as above (Yao *et al.* ▸, in the press). Moreover, the same study formulated a model combining the modulation parameter γ and *d*_sub_ to predict the upper (Bi-rich) end of the tetradymite series.

Study of layered compounds in the system PbTe–Bi_2_Te_3_ led to the definition of another homologous series based on units of fixed width: *n*PbTe·*m*Bi_2_Te_3_ (Shelimova *et al.*, 2004 ▸) using a similar approach to the definition of *n*Bi_2_·*m*Bi_2_Te_3_ for compounds in the tetradymite series (Shelimova *et al.*, 2000 ▸). Following the same ideas, Kuribayashi *et al.* (2019 ▸) discovered and named the third member of the aleksite series, hitachiite (Pb_10_Bi_4_Te_4_S_12_), and introduced the formula Bi_2_Te_2_S·*n*PbS to express homology in the series, an approach distinct from the accretional model described above.

*Ab initio* calculations of phases across an extended compositional range in a modular series provide an excellent tool for the exploration of modularity, crystal structures, phase stabilities, and the limits of the series. Using density functional theory (DFT) and structure simulations we study seven homologues from the aleksite series covering the compositional range Bi_2_Te_2_S–Pb_12_Bi_4_Te_4_S_14_. Our objectives are: (i) to describe their structures, bonding, structural–chemical modulation and phase stabilities, (ii) build a model for predicting the upper (Bi-rich) limit of the series and (iii) discuss similarities and differences between the aleksite and tetradymite series.

## 2. Crystal structure data and selection of input files

**Figure 1:**
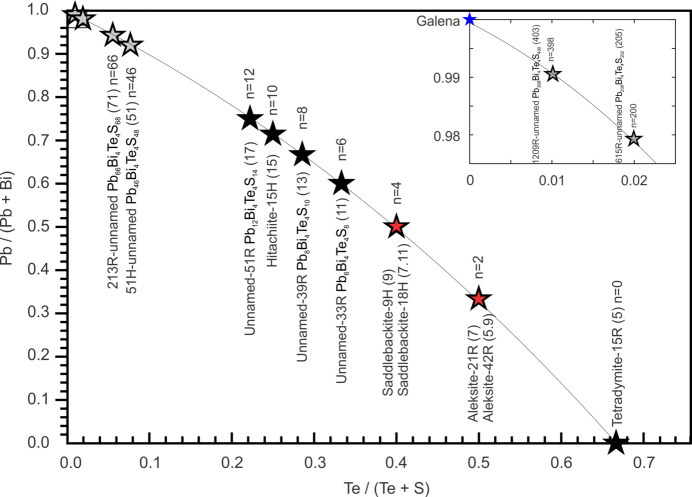
Plot of Pb/(Pb+Bi) versus Te/(Te+S) indicating compositions of phases in the aleksite series. The seven single-module structures (5-, 7-, 9-, 11-, 13-, 15- and 17-atom layers) are indicated by large black stars and the two corresponding double-layer polytypes 5.9 (7-), 7.11 (9-) are represented by filled red stars. Theoretical phases close to the PbS end of the series with 51-, 71-, 205- and 403-atom layers are shown by grey stars. The latter are also plotted together with galena (blue star) in the inset figure, for clarity.

Table 1 ▸ lists the seven phases under investigation (four minerals and three unnamed phases) and published data relating to their crystal structures. These are also shown on a diagram of (Pb/Pb+Bi) versus Te/(Te+S) (Fig. 1 ▸). They represent seven discrete homologues with even-numbered values of *n* (0, 2, 4, 6, 8, 10 and 12) using the formula Pb_*n*_Bi_4_Te_4_S_*n*+2_ given by Cook *et al.* (2007*a* ▸, 2019 ▸). The corresponding structures are given by the formula *S*(*M*_*p*_ *X*_*p*+1_)·*L*(*M*_*p*+1_ *X*_*p*+2_), where *p* = 2, *S* = the 5-atom layer (for simplicity termed ‘5-layer’ hereafter), and six different *L* modules (7-, 9-, 11-, 13-, 15- and 17-layers). In addition, homologues *n* = 2 and *n* = 4, corresponding to the minerals aleksite (7-layer) and saddlebackite (9-layer), respectively, are each represented by simple, double-module polytypes [aleksite-42R with the stacking sequence (5.9), and saddlebackite-18H with (7.11) stacking sequence]. The aleksite-54H polytype (Spiridonov, 1995 ▸), with much longer stacking sequence (77.11.77.15), was not included due to the much longer computation time required.

All phases are trigonal, but the space group changes from 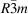 (R) to 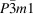 (H) whenever the total number of atoms in the explicit formula is divisible by 3. The total number of layers in each structure is *N* = *N*_1_ × 3 for R phases and *N* = *N*_1_ × 1 in H. The *d*_sub_ value is calculated from experimental data using *c*/*N*_total._ In the unnamed phases, the *c* parameter is calculated assuming an interlayer distance of *d*_0_ ∼ 2 Å. We found that *a* remains constant at around 4.23 Å whereas *c* shows large variation depending on the space group and the stacking sequence of individual polytypes. Nevertheless, their interlayer distances (*d*_sub_ = *c*/*N*_total_) are directly comparable with one another and decrease systematically with increasing Pb and S content.

## 3. Methods

### 3.1. Ab *initio* calculations

To understand the connection between crystal structure and chemistry in aleksite mixed layer compounds, we performed *ab initio* total energy calculations and structure relaxations based on density functional theory (DFT) (Hohenberg & Kohn, 1964 ▸; Kohn & Sham, 1965 ▸). We used the VASP simulation package (Kresse & Furthmüller, 1996 ▸) based on the projector augmented wave (PAW) method (Blöchl, 1994 ▸). The exchange and correlation energy are treated with the generalized gradient approximation (GGA) within the Perdew, Burke and Ernzerhof (PBE) scheme (Perdew *et al.*, 2008 ▸). The gamma-centered dense *k* points were used to sample the Brillouin zone (Table 2 ▸) and plane waves are expanded at cutoff energy 600 eV. van der Waals interactions (Te–Te) were included using the method of Grimme *et al.* (2010 ▸). Structures were relaxed with energy convergence of less than 10^−6^ eV for each ionic step and forces on each atom are within 0.02 eV Å^−1^.

In order to obtain the equation of state (EOS) for each structure, relaxations were carried out at different volumes with lattice vectors scaled from 95 to 101%. The relationship between volume and total energy was fitted using the Murnaghan (1944 ▸) equation of state:

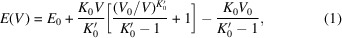

where *K*_0_ and 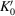 are the bulk modulus and its pressure derivative, *V*_0_ is the equilibrium volume and *E*_0_ is the reference energy. For each structure, the relaxed unit-cell parameters are obtained by calculating structure relaxations at equilibrium volume.

Upon completing the structure relaxations for each phase, we calculate the formation energy (Δ*E*_f_) to evaluate the relative phase stability. Applying a similar approach to that used by Woodcox *et al.* (2019 ▸), we establish a simple relation between Δ*E*_f_, the energy for each phase (*E*_phase_) and the energy of single atoms (*E*_Bi_, *E*_Te_, *E*_Pb_ and *E*_S_) in equation (2):

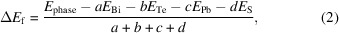

where *a*, *b*, *c* and *d* represent the number of atoms of Bi, Te, Pb, and S, respectively, within each structure. When Δ*E*_f_ ≤ 0, the phase is considered potentially stable. An alternative approach to establishing the relative stability of a phase considers the energy difference to endmembers (Park *et al.*, 2021 ▸), *i.e.* tetradymite (Bi_2_Te_2_S) and galena (PbS), using equation (3):

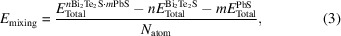

where 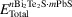 , 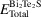 and 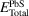 are the total energies of each mixed phase and *N*_atom_ = total number of atoms.

## 4. Results

### 4.1. Crystal structure relaxation

**Figure 2:**
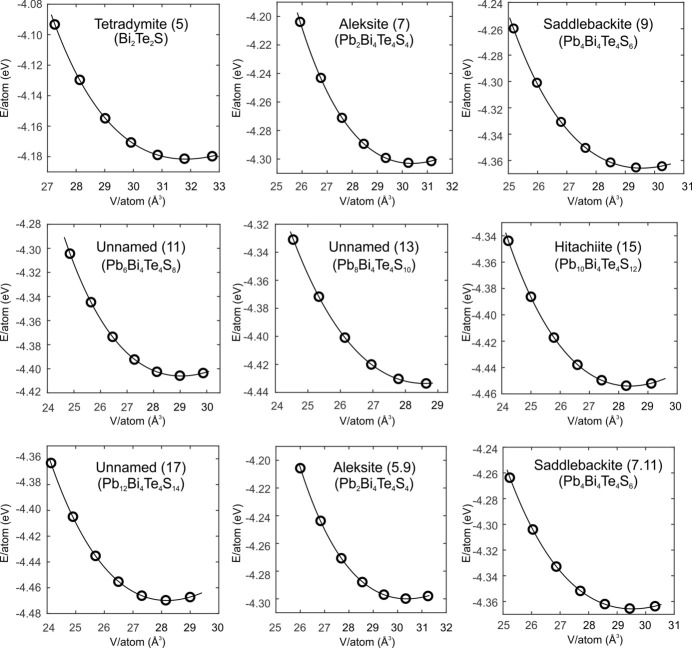
Total energy curves of the nine structures corresponding to seven phases in the aleksite series as a function of the atomic volume. Open black circles represent DFT-calculated total energies. Solid black lines are determined by fitting the Murnaghan equation of state (1). Seven of these are single-module structures *n* = 0, 2, 4, 6, 8, 10, 12, and two are the double-layer polytypes of aleksite (5.9) and saddlebackite (7.11).

We adopted the experimental *a* and *c* unit-cell parameters in Table 1 ▸ as input for the total energy calculations. The same procedure used by Yao *et al.* ▸ (in the press) was applied to obtain the initial atomic coordinates for the *N* number of atoms in each crystal structure. The *z* coordinates are at equal intervals of 1/*N* along *c*, and the corresponding *x*, *y* coordinates are at 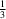 , 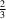 and 0 values repeating for a group of three atoms. To obtain the relaxed structures, we firstly constrain the equilibrium volume for each phase by fitting the total energy volume curves (Fig. 2 ▸) using the Murnaghan equation of state with EOS parameters tabulated in Table 3 ▸. The calculated *V*_0_ values agree with published data (Table 3 ▸) within 3.6% for all available structures.

**Figure 3:**
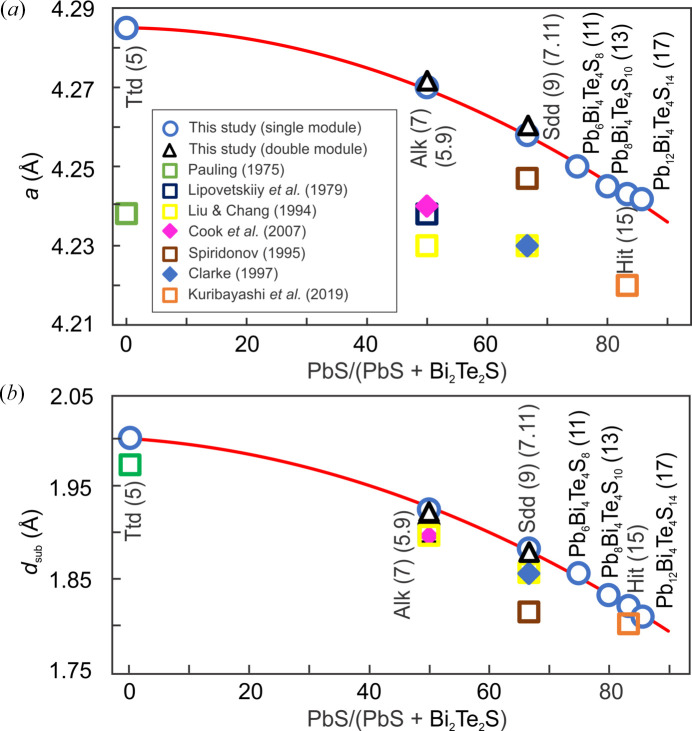
Variation in (*a*) unit-cell parameter *a* and (*b*) interlayer distance *d*_sub_ plotted as a function of PbS/(PbS+Bi_2_Te_2_S) for the seven studied phases. Published experimental data (Table 1 ▸) are included for comparison. Ttd is tetradymite, Alk is aleksite, Sdd is saddlebackite, Hit is hitachiite.

The final structure parameters are obtained from the DFT calculations at the *V*_0_ values for all phases. The relaxed *a* and *c* unit-cell parameters are within 1.5% difference with the published data (Tables 1 ▸ and 4 ▸). Comparison with experimental data shows a slight overestimation in the *a* parameter [Fig. 3 ▸(*a*)] and a good fit for *d*_sub_ values [Fig. 3 ▸(*b*)]. Both *a* and *d*_sub_ parameters show a smooth decreasing trend with increasing PbS across the compositional interval investigated. Notably, the double-module polytypes of aleksite and saddlebackite yield values for *a* and *d*_sub_ that are very similar to those of their respective single-module polytypes (Fig. 3 ▸).

### 4.2. Crystal structure models

**Figure 4:**
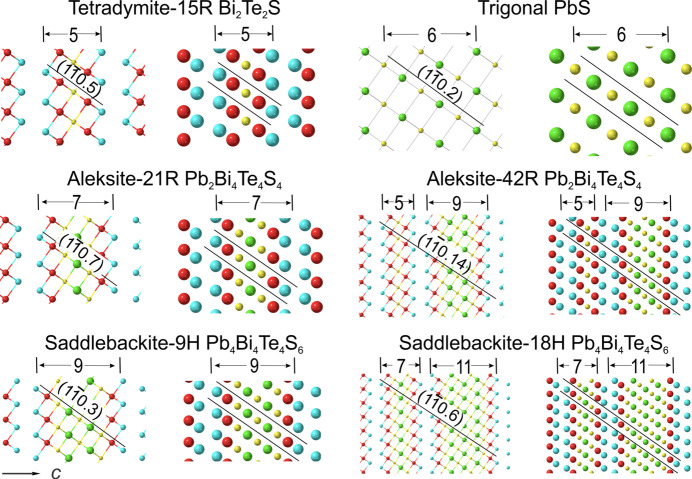
Crystal structure models (ball and stick on left, atom filling on right) for the relaxed structures of phases where *n* = 0, 2 and 4 on 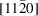 zone axis. Trigonal PbS from literature is also shown. Layer stacks and their corresponding widths are labelled on the top of each. Atom arrangements (red = Bi, blue = Te, yellow = S and green = Pb) depicting the structure are plotted along the (*hkil*) planes, *i* = −(*h*+*k*). A crystallographic information file (CIF) is provided in the supporting information.

The crystal structure models obtained using the relaxed unit-cell parameters are plotted on the zone axis 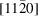 to illustrate the incremental increase in width of each structure with addition of Pb and S atoms (Figs. 4 ▸ and 5 ▸). We note that the building modules are centred onto a slab of S–Pb–S…Pb–S flanked on each side by Bi–Te atoms. The increment of the central slab can be expressed as: Pb_*k*_S_*k*+1_ (*k* = 0–6) for the six homologues discussed here. The modules are always separated by Te–Te layers (van der Waals gaps). A trigonal PbS structure (PbS_R_) obtained by transformation from cubic galena (Noda *et al.*, 1987 ▸) is included for comparison (Fig. 4 ▸). This shows the atomic arrangement in PbS_R_ is very similar to the central slab in aleksite structures when viewed on the 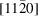 zone axes.

The models show that the tetradymite unit is no longer preserved as such within the single-module structures, although these are required to form all homologues with *n* < 2 such as the unnamed *n* = 1 phase with composition PbBi_4_Te_4_S_3_ (Cook *et al.*, 2019 ▸). Additionally, 5-atom tetradymite modules are constituent building blocks in the double-module aleksite polytype, aleksite-42R (*n* = 2) considered in this contribution, which contains a (5.9) sequence (Fig. 4 ▸). Five-atom tetradymite modules may potentially exist in other configurations with *n* > 2 (*e.g.* 5.13 saddlebackite), although these will not be considered here. Alongside single-module 9H saddlebackite (*n* = 4), we do, however, consider the 18H double-module polytype structure of saddlebackite, which features a (7.11) sequence. We note that the simplest polytypes representing the *n* = 2*k*+2 (*k* = integer) building modules are composed of two single-module units with *n* = 2*k* and *n* = 2*k* + 4.

Slight lattice distortion is observed within structures containing two different modules, *e.g.* the shift between the 5- and 9-atom layer stacks in the aleksite-42R polytype. Variations in atom arrangements within the single-module structures are, in contrast, negligible.

### 4.3. Bond analysis

**Figure 6:**
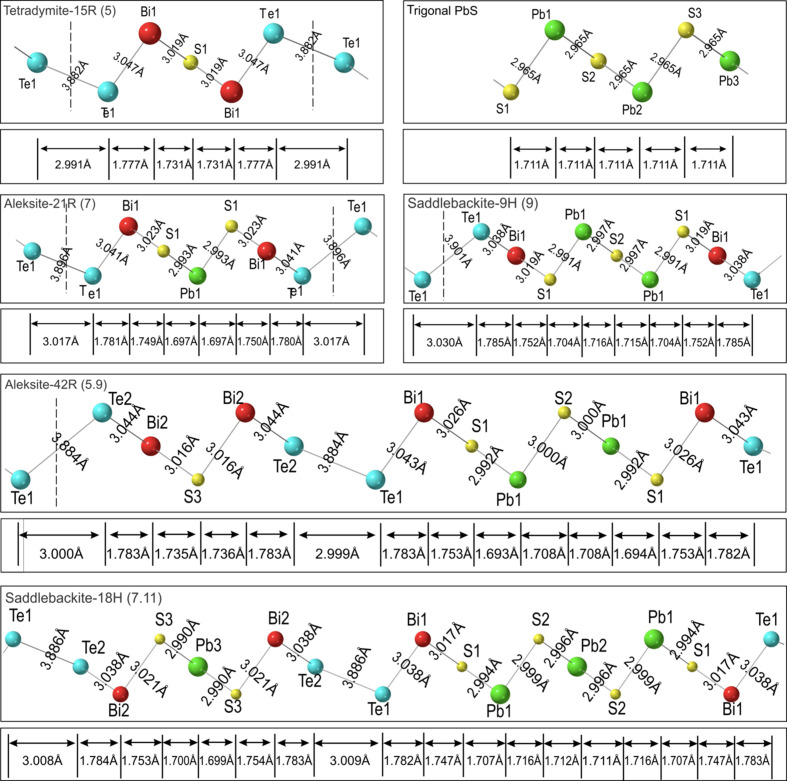
Bond types and bond lengths for the relaxed structures of phases where *n* = 0, 2 and 4 within the asymmetric unit cell plotted on the 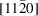 zone axis. Trigonal PbS from the literature is also illustrated for comparison. Red = Bi, blue = Te, yellow = S, green = Pb. Projections of bonds along the *c* axis are labelled underneath, they represent the bond length contributions to *d*_sub_ shown in Fig. 8 ▸.

The bond types and their variation in length across the studied phases are shown within the asymmetric unit cell for all structures (Figs. 6 ▸ and 7 ▸). The three types of bonds in tetradymite (Bi—Te, Bi—S and Te—Te) are complemented by Pb—S bonds in all other compounds from the aleksite series. The Bi—Te bond decreases in length from tetradymite (3.047 Å) to the 17-atom layer structure (3.036 Å) whereas the length of the Bi—S bond remains relatively constant at ∼3.019 Å. Likewise, Te—Te bond lengths increase from 3.882 Å in tetradymite to a maximum of 3.919 Å in the 11-atom layer. The Te—Te bonds in the two aleksite polytypes are constant and close to those in tetradymite (3.884 Å and 3.886 Å for the 5.9- and 7.11-atom layer sequences, respectively). Although the average Pb—S bond length is nearly constant ∼2.990 Å, there is a small variation within the middle Pb_*k*_S_*k*+1_ slab, *e.g.* from 2.986 to 2.995 Å in the 17-atom layer. The Pb—S bond lengths within the aleksite and saddlebackite double-module polytypes are nearly identical to those in the corresponding single-module polytypes.

In Figs. 6 ▸ and 7 ▸, bond lengths are projected onto the *c* axis to calculate the contribution towards the *d*_sub_ value in each structure. Te—Te bond projections have the highest values on the *c* axis whereas Bi–Te and Bi–S projections are only slightly larger than Pb—S bond projections. In all single-module structures, there is one Te—Te bond, two Bi—Te bonds and two Bi—S bonds, whereas the number of Pb—S bonds increases from 0 in tetradymite to 12 in the 17-atom layer, with an incremental step of 2. We thus divide the bond types into two groups: variable number (Pb—S) and fixed number (Bi—Te, Bi—S and Te—Te).

**Figure 8:**
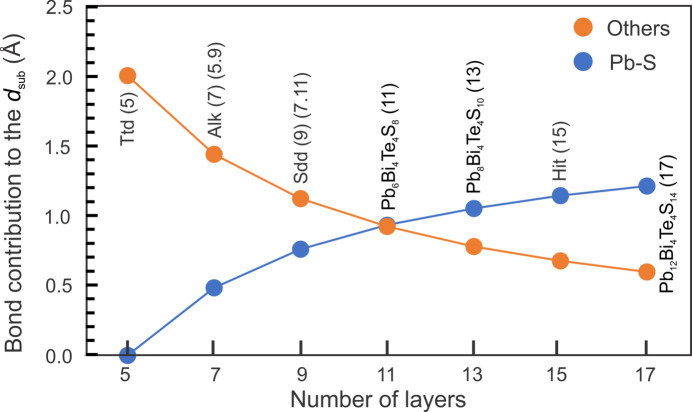
Bonds length contributions to *d*_sub_ for the seven single-module phases (*n* = 0, 2, 4, 6, 8, 10 and 12). The Pb—S contribution is shown in blue, and others (Bi—Te, Bi—S and Te—Te) are shown in orange. The two types of bonds display contrasting trends: the contribution from Pb–S increasing from the 5- to 17-atom layer, whereas the contribution of other bonds systematically decreases.

We have calculated the bond contribution to the *d*_sub_ parameter from cumulative projection values and their abundance across the compositional range studied (Fig. 8 ▸). This plot shows two opposing trends, an increase in Pb—S contribution and decrease in contribution from other bonds from tetradymite to the 17-atom layer, the two lines intersecting at the 11-atom layer. The increase in the contribution to *d*_sub_ from the Pb—S bonds is however more moderate than the contribution decreases from the other bonds, resulting in a modest decrease of *d*_sub_ with increased PbS concentration [Fig. 3 ▸(*b*)].

### 5.4. STEM simulation and electron diffraction

**Figure 9:**
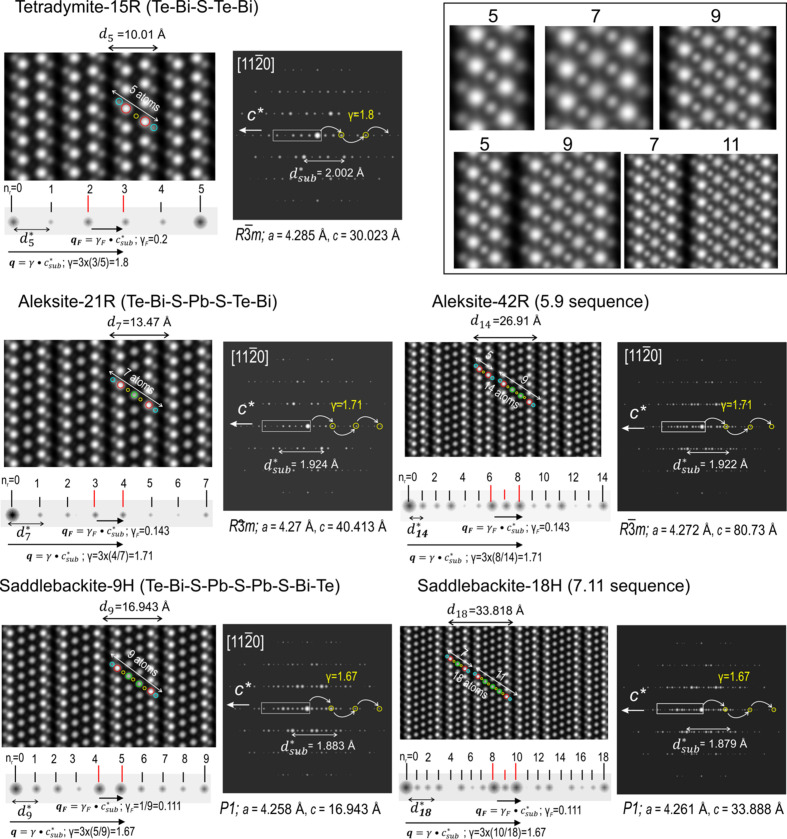
STEM simulations (left) and corresponding electron diffraction (ED) patterns (right) for the relaxed structures of tetradymite (15R), aleksite (21R and 42R) and saddlebackite (9H and 18H) shown on the 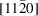 zone axis. Unit-cell parameters *a* and *c* and the interlayer distance *d*_sub_ for each structure are tabulated in Table 4 ▸. The ribbon below each STEM image is cropped from the ED patterns, showing the number of reflections and their intensity variations along the 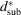 interval. Two modulation vectors (**q** and **q**_*F*_) underpinning structural modulation are marked by arrows. The atom layer arrangement for each structure is marked by circles (cyan = Te, red = Bi, yellow = S and green = Pb). Layer stacks within all structures are placed top right on the figure. Note that simulations for saddlebackite (9H and 18H) were carried out with space group *P*1 rather than 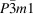 .

**Figure 10:**
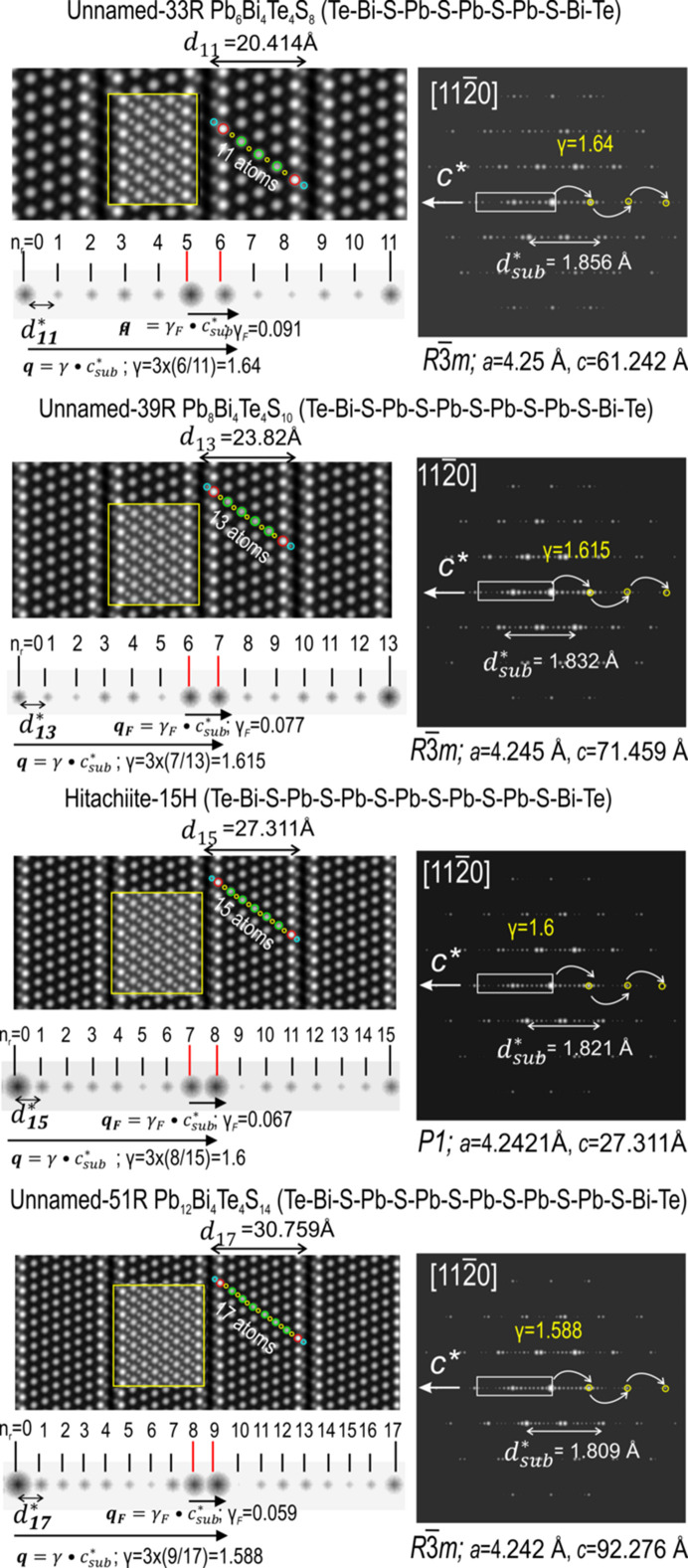
STEM simulations (left) and corresponding electron diffraction (ED) patterns (right) for the relaxed structures of Pb_6_Bi_4_Te4S_8_ (33R), Pb_8_Bi_4_Te_4_S_10_ (39R), hitachiite (15H) and Pb_12_Bi_4_Te_4_S_14_ (51R) shown on the 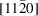 zone axis. Unit-cell parameters *a* and *c* and the interlayer distance *d*_sub_ for each structure are tabulated in Table 4 ▸. The ribbon below each STEM image is cropped from the ED patterns, showing the number of reflections and their intensity variations along the 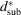 interval. Two modulation vectors (**q** and **q**_*F*_) underpinning structural modulation are marked by arrows. The atom layer arrangement for each structure is marked by circles (cyan = Te, red = Bi, yellow = S and green = Pb). Layer stacks within all structures are marked by the overlays on the images. Note that simulations for hitachiite (15H) were carried out with space group *P*1 rather than 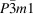 .

In Figs. 9 ▸ and 10 ▸ we show the relaxed structures in STEM simulations and electron diffraction (ED) patterns on zone axis 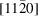 . The signal intensity (*I*) in (HAADF) STEM imaging is proportional to *Z* ^2^ of an element along an atomic column (Pennycook & Jesson, 1990 ▸; Ishizuka, 2002 ▸). We note that S (*Z* = 16) is not displayed on the simulations when the neighbouring atom columns are Bi (*Z* = 83) and Pb (*Z* = 82). A better visualization of the number of atoms in each structure can be assembled using Se (*Z* = 34) instead of S (inset, top right in Fig. 9 ▸ and overlays on each STEM simulation in Fig. 10 ▸). Such simulations agree very well with the atomic arrangement models displayed in Figs. 4 ▸ and 5 ▸ and are concordant with HAADF STEM images of phases from the aleksite series (Cook *et al.*, 2019 ▸).

The 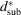 interval (cropped from the ED patterns) is essential for constraining structural modulation in terms of the increase in module width and module combinations. Within each 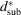 interval, there are *N*_1_ − 1 number of reflections equally distributed. The asymmetric unit-cell length ( 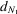 ) is correlated with layer stacks for each structure. This can be also indicated as the smallest interval, 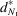 between two neighbour reflections along 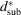 .

Typical of all phases in the series is the fact that the ED patterns show the two brightest reflections at the centre of 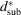 . This interval, underpinned by the modulation vector **q**_*F*_ = 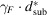 , where γ_*F*_ = *i*/*N*_1_·*d*_sub_ ∼ 1/*N*_1_; *i* = *S* + *L*. γ_*F*_ values are within the range 0.2–0.059 for the analysed structures and this shows a monotonic decrease with increase in PbS concentration. In cases where there are multiple polytypes, although the number of divisions is doubled relative to their single unit structure, *e.g.* 14 and 7 divisions for the 42R and 21R aleksite polytypes, the **q**_*F*_ vector remains unchanged. Nonetheless, the **q**_*F*_ interval is split into two by a satellite reflection of lesser intensity (Fig. 9 ▸). On the other hand, the displacive modulation between chalcogen (S, Te, Se) and Pb and Bi atoms is underpinned by a second vector: **q** = 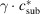 (Lind & Lidin, 2003 ▸). The **q** modulation is depicted up to third-order reflections along c* (ED patterns in Figs. 9 ▸ and 10 ▸). Values of γ (1.8–1.588 for the 5- to 17-atom layer module range) are calculated as 3[(*N*_1_ + 1)/2]/*N*_1_ for single modules. In the double-module polytypes γ = 3[(*N*_1_ + 2)/2]/*N*_1_ giving the same values of γ as the corresponding single-module structures (Fig. 9 ▸).

This formalism is in agreement with the crystal structural formula: *S*(*M*_*p*_ *X*_*p*+1_)·*L*(*M*_*p*+1_ *X*_*p*+2_), *X* = chalcogen, where *S* and *L* are the number of shorter and longer modules (Cook *et al.*, 2019 ▸) but not the formula *n*PbTe·*m*Bi_2_Te_3_ of Shelimova *et al.* (2004 ▸). For example, aleksite-21R would have *n* = *m* = 1, requiring two distinct modules instead of only one. The 42R polytype will have 4 modules (*n* = *m* = 2) instead of the ‘5’ and ‘9’ modules considered here. Such a strong correlation between electron diffraction patterns and chemical modules in a homologous series is typical for mixed layer compounds (Amelinckx *et al.*, 1989 ▸).

## 5. Discussion

### 5.1. Phase stability and energy mixing

Formation energies [equation (2)] for the studied phases in the interval Bi_2_Te_2_S–Pb_12_Bi_4_Te_4_S_14_ (*n* = 12) are given in Table 5 ▸. Calculation of the formation energy and energy of mixing requires the DFT reference energies (*E*_0_) of all elements (Bi, Pb, Te and S) and endmembers (Bi_2_Te_2_S and PbS). The reference energies for Pb, S, Bi_2_Te_2_S (Table 3 ▸), and PbS are calculated from equation of state fitting [equation (1)] in this study, those for the elements Bi and Te are adopted from Yao *et al.* ▸(in the press). Their parameters are summarized in Table 6 ▸. All reference energies are calculated based on the GGA functional.

The calculated Δ*E*_f_ values are negative for all nine phases and decrease as the PbS component increases, implying they are relative stable to the endmembers. The larger double-module polytypes of both aleksite (42R) and saddlebackite (18H) show the same formation energy as their corresponding single-module units (21R and 9H, respectively), implying they are equally stable.

**Figure 11:**
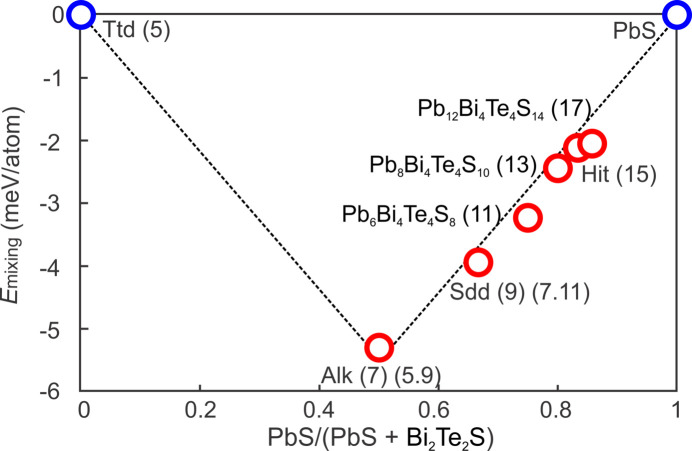
Phase stability of aleksite series phases evaluated from a plot of energy of mixing versus PbS/(PbS+Bi_2_Te_2_S). The plot features a convex Ttd–Alk–PbS hull. The phases Sdd, Pb_6_Bi_4_Te_4_S_8_, Pb_8_Bi_4_Te_4_S_10_, Pb_10_Bi_4_Te_4_S_12_, Hit and Pb_12_Bi_4_Te_4_S_14_ all lie below the Alk–PbS part of hull, indicating they can be stable. The two endmembers, tetradymite and PbS, are represented by blue circles; the seven aleksite series phases are represented by red circles. Ttd = tetradymite, Alk = aleksite, Sdd = saddlebackite, Hit = hitachiite.

Phase stability can also be evaluated from the energy of mixing (*E*_mixing_), which is calculated using values of the two endmember phases, tetradymite and galena [equation (3)]; Table 5 ▸). This defines a convex hull between tetradymite and galena (PbS) with aleksite at the lowest energy point (Fig. 11 ▸). The other five studied homologues plot along or slightly below the branch between aleksite and galena. Such a distribution indicates that all studied phases can be relative stable compared with the endmembers and thus do not readily decompose into tetradymite and galena endmembers. However, whether the studied phases are thermodynamically stable may require further phonon calculations to investigate the thermal effects and entropy contributions (*e.g.* Belmonte *et al.*, 2014 ▸).

Instead of adopting the formula *n*PbS·*m*Bi_2_Te_2_S as a working model, the energy of mixing can also be defined using the accretional model:

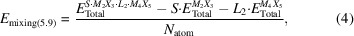




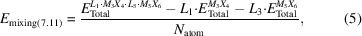

where *S* = 5-atom layer, *L*_1–3_ represent longer 7-, 9- and 11-modules; *M* = Bi, Pb, and *X* = Te, S. The energy of mixing for aleksite-42R (5.9) and saddlebackite-18H (7.11) are found at 0 and 0.2 meV per atom, respectively. This shows ideal mixing when using the accretional model and indicates that the derived polytypes and, indeed, other multiple-module structures in the series can be stable relative to their single-module components. Further calculations may, however, be required to fully validate these findings.

### 5.2. The γ–*d*_sub_ relationship: a model for the extension of the aleksite series

Preliminary work shows that homologues of the aleksite series with still greater PbS content (*n* = 18 and *n* = 30, representing 23- and 35-atom layers, respectively) are present in assemblages buffered by galena (Cook *et al.*, 2021 ▸ and unpublished data). Theoretical phases from the PbS-rich end of the series, such as 403-, 205-, 71- and 51-atom layers (corresponding to homologues with *n* = 398, 200, 66 and 46), can also be considered based on their chemistry, which is close to, but distinct from, PbS (Fig. 1 ▸).

**Figure 12:**
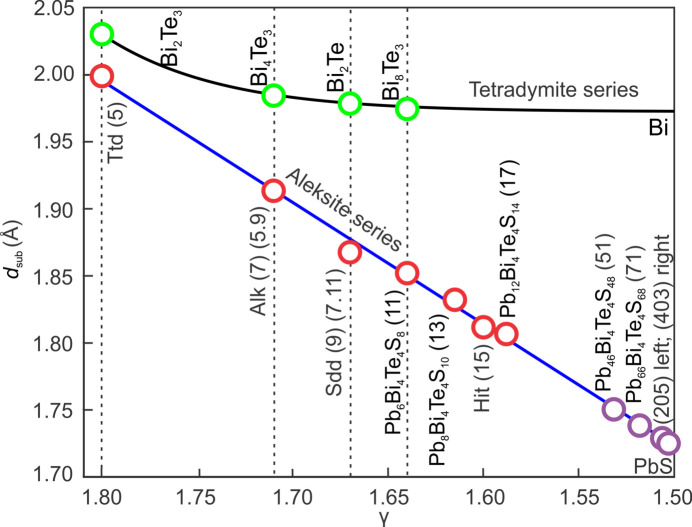
Model of the relation between γ and *d*_sub_ for the aleksite series (red circles, solid blue line, this study) compared with the tetradymite series (green circles, solid black line) from Yao *et al.* ▸ (in the press). The linear curve for the aleksite series is fitted from the γ and *d*_sub_ values of the seven single-module phases. This allows extrapolation to theoretical phases (purple circles) the end of the series near PbS. Phases with the same γ values from the two series are highlighted by dashed lines.

Our model describes a quasilinear relationship between γ and *d*_sub_ (Fig. 12 ▸), which allows the prediction of *d*_sub_ for any phase across the 17- to 403-atom layer structure range (γ = 1.588–1.504), with *d*_sub_ values over this interval lying in the range 1.806 to 1.726 Å. The theoretical 403-atom layer phase, Pb_398_Bi_4_Te_4_S_400_, with Pb/(Pb + Bi) = 0.99 shows identical *d*_sub_ values as our DFT-modelled predictions for PbS_T_, which is also within 0.8% difference of that for trigonal PbS transformed from the experimental cubic structure (Noda *et al.*, 1987 ▸). As a result, our model is suitable to approximate *d*_sub_ values for aleksite series homologues across the entire compositional range from tetradymite to the PbS_T_ endmember.

### 5.3. Modularity and comparison with the tetradymite series

Noting the possibility of multiple polytypes for many, if not all, homologues in the aleksite series (Cook *et al.*, 2019 ▸), we introduce a modified formula:

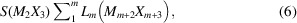

where *S* represents the number of 5-atom layers, and *L*_1_, *L*_2_,…*L*_*m*_ are the numbers of longer, 7-, 9-,…2*m* + 5 modules; *m* > 0, integer; and *S*, *L* ≥ 0. This formula is useful for expressing the range of polytypes for each homologue within the series. Therefore, applying formula (6) to Pb_*n*_Bi_4_Te_4_S_*n*+2_ from Cook *et al.* (2007*a* ▸, 2019 ▸), we can calculate the homologue number (*n*) by relating the total number of cations and chalcogens within the component modules:

*n* + 4 = 2*S* + 3*L*_1_ +…(*m* + 2)*L*_*m*_, leading to *n* = 2*S* + 3*L*_1_ +…(*m* + 2)*L*_*m*_ − 4 for the number of cations, and *n* + 6 = 3*S* + 4*L*_1_ +…(*m* + 3)*L*_*m*_, leading to *n* = 3*S* + 4*L*_1_ +…(*m* + 3)*L*_*m*_ − 6 for the number of chalcogens.

The theory of mixed layer compounds stipulates that structures built by modules which are distinct in size and chemical composition are related to one another by characteristics of electron diffraction patterns thus underpinning the modularity within a homologous series (Amelinckx *et al.*, 1989 ▸). Both the aleksite and tetradymite series are formed by modular structures derived from the same 5-atom archetype but with distinct compositional ranges, *i.e.* extending towards PbS (aleksite series) and Bi endmembers (tetradymite series).

The individual building modules in each series are composed of an uneven number of atoms, 7, 9, 11…2 *k*+ 1, but with different topology between cations (Bi, Pb) and chalcogens (Te, S, Se), *i.e.*, symmetrical in the aleksite series and asymmetrical in the tetradymite series. Despite this, the electron diffractions of relaxed structures from the aleksite series (Figs. 9 ▸ and 10 ▸) show identical modulation vectors as corresponding phases in the tetradymite series with the same number of layers and/or building modules (Ciobanu *et al.*, 2009 ▸; Yao *et al.* ▸, in the press). Such characteristics provide a strong link between the two series and prove their affiliation to a single class of mixed layer compounds built by the same accretional homology principles. The alternative homology proposed for the two series involving units of the same size, 2- and 5-atom layers (Shelimova *et al.*, 2000 ▸, 2004 ▸; Kuribayashi *et al.*, 2019 ▸) is not supported by the crystal structures, even though it may be conceptually useful to depict chemical variation within each of the two series.

Bond analysis shows marked differences between the two series, whereby the longer Te—Te bonds are present in all homologues of the aleksite series and may be responsible for the extensive polytypism. In contrast, the Te—Te bonds are only present in Te-rich members of the tetradymite series (Yao *et al.*, ▸ in the press). Construction of incremental symmetrical modules by addition of Pb–S in the aleksite series and by asymmetrical modules involving Bi–Bi pairs in the tetradymite series leads to linear versus non-linear features in the respective γ–*d*_sub_ relationships. As a result, for the same γ interval (1.8–1.5) the range of *d*_sub_ is greater for the aleksite series compared with the tetradymite series, *i.e.* ∼2 to 1.726 Å, and ∼2 to 1.973 Å, respectively (Fig. 12 ▸).

## 6. Conclusions and implications

The crystal structures and stabilities of phases from the aleksite homologous series, Pb_*n*_Bi_4_Te_4_S_*n*+2_, where *n* = homologue number (Cook *et al.*, 2019 ▸), were calculated using DFT methods. The study addressed four named minerals (tetradymite, aleksite, saddlebackite and hitachiite) and three compounds yet to be described in natural specimens (Pb_6_Bi_4_Te_4_S_8_, Pb_8_Bi_4_Te_4_S_10_ and Pb_12_Bi_4_Te_4_S_14_). The seven phases represent homologues where *n* = 0, 2, 4, 6, 8, 10 and 12. Each homologue corresponds to a single-module type with an uneven number of atoms (5, 7, 9, 11, 13, 15 and 17, respectively), expressed by the formula: *S*(*M*_*p*_ *X*_*p*+1_)·*L*(*M*_*p*+1_ *X*_*p*+2_), where *M* = Pb, Bi, and *X* = Te, S), *p* ≥ 2, *S* = five-atom layer, and *L*1–6 = 7, 9, 11, 13, 15 and 17-atom layers. The *n* = 2 and *n* = 4 homologues are also represented by two-layer polytypes (aleksite-42R and saddlebackite-18H), which have structures comprising two differently sized modules, (5.9) and (7.11), respectively. Other multi-layer polytypes are predicted to exist for phases across the series.

The relaxed structures show the unit-cell parameters *a* and *c* within 1.5% of available experimental data. Both *a* and the interlayer distance *d*_sub_ show decrease with increasing PbS component in the relaxed structures. Crystal structure models and STEM simulations show that the six single modules (for structures with *n* > 0) are centred onto a Pb_*k*_S_*k*+1_ slab (*k* = 1–6), with S–Pb–S…Pb–S arrangement flanked by Bi–Te atoms. We show variable Pb—S bond lengths in the aleksite homologues, representing an important structural difference compared to the constant Pb—S bond lengths in galena.

Electron diffraction patterns show *N*_1_ intervals of equal length along 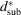 demonstrating that all phases are *n*-fold superstructures of a rhombohedral subcell with *c*/3 = 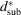 . The modulation vector **q** = 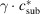 shows a decrease in γ, from 1.8 to 1.588, with increasing PbS component across the compositional range studied (*n* = 0 to 12). The ED patterns have two brightest reflections at the centre of 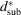 , which are described by the modulation vector **q**_*F*_ = 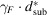 (γ*_F_* = 0.2–0.059). The number of divisions within this central interval corresponds to the number of modules, *i.e.* 1 for single, and 2 for double modules. This result proves that the homologous structures can be described by the formula *S*(*M*_*p*_ *X*_*p*+1_)·*L*(*M*_*p*+1_ *X*_*p*+2_), and not the formula *n*PbS·*m*Bi_2_Te_2_S, involving 2- and 5-atom building units (Shelimova *et al.*, 2000 ▸).

The DFT method is also used to obtain the formation energies and energy of mixing for all seven compositions. The seven single-module structures and the two double-module polytypes show negative formation energies, implying they can be relative stable to their endmembers.

We established a linear γ and *d*_sub_ model which allows the calculation of *d*_sub_ for any phase beyond the compositional range studied, *e.g.* phases with *n* values of 46, 66, 200 and 398. The model predicted a *d*_sub_ value of 1.726 Å for the phase Pb_398_Bi_4_Te_4_S_400_ (*n* = 398). This can be considered as the upper end of the series, as this is the same value obtained for PbS_T_ in DFT calculations, and lies within 0.8% of experimental data.

The aleksite and tetradymite series represent excellent examples of mixed layer compounds built by accretional homology principles derived from a shared 5-atom layer archetype. This study illustrates how DFT calculations can not only support predictive models for crystal and chemical modularity, but also represent a tool to expand and ultimately constrain the limits of modular series. Potential applications exist to model other, chemically different, mixed layer structures. 

## Supplementary Material

Crystal structure: contains datablock(s) global, I, II, III, IV, V, VI, VII, VIII, IX. DOI: 10.1107/S2052520623008776/dk5122sup1.cif

CCDC references: 2302229, 2302230, 2302231, 2302232, 2302233, 2302234, 2302235, 2302236, 2302237

## Figures and Tables

**Figure 5:**
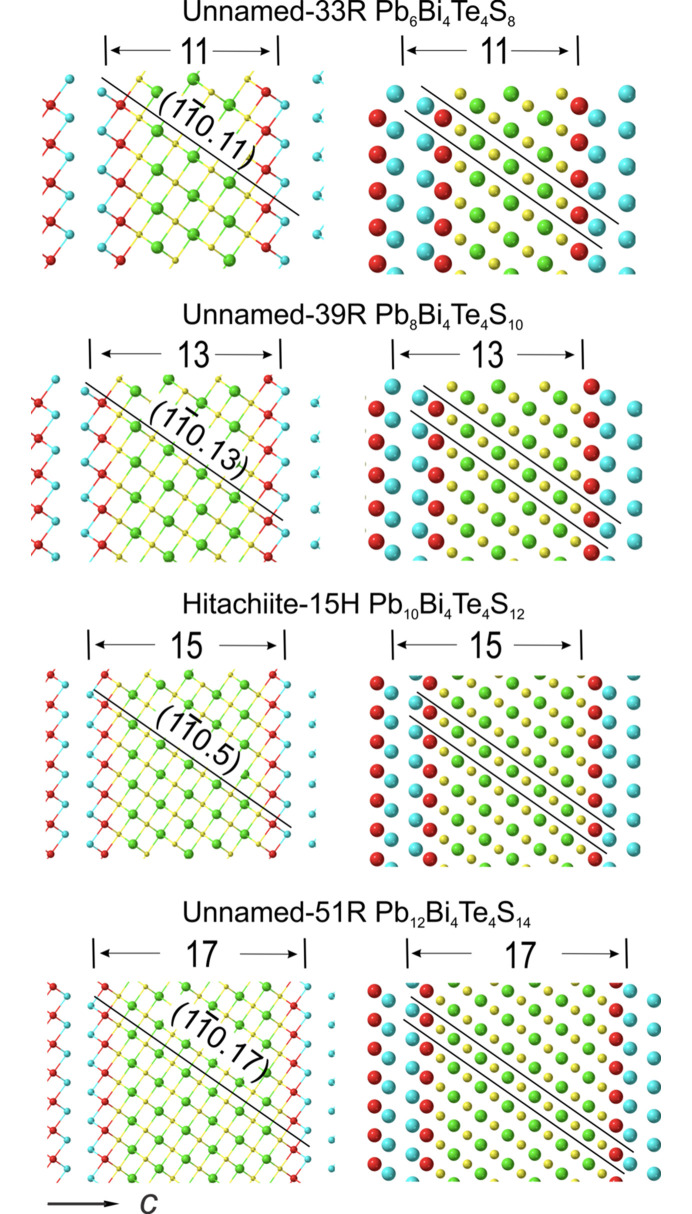
Crystal structure models (ball and stick on left, atom filling on right) for the relaxed structures of phases where *n* = 6, 8, 10 and 12 on 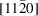 zone axis. Layer stacks and their corresponding widths are labelled on the top of each. Atom arrangements (red = Bi, blue = Te, yellow = sulfur, green = Pb) depicting the structure are plotted along the (*hkil*) planes, *i* = −(*h*+*k*). A crystallographic information file (CIF) is provided in the supporting information.

**Figure 7:**
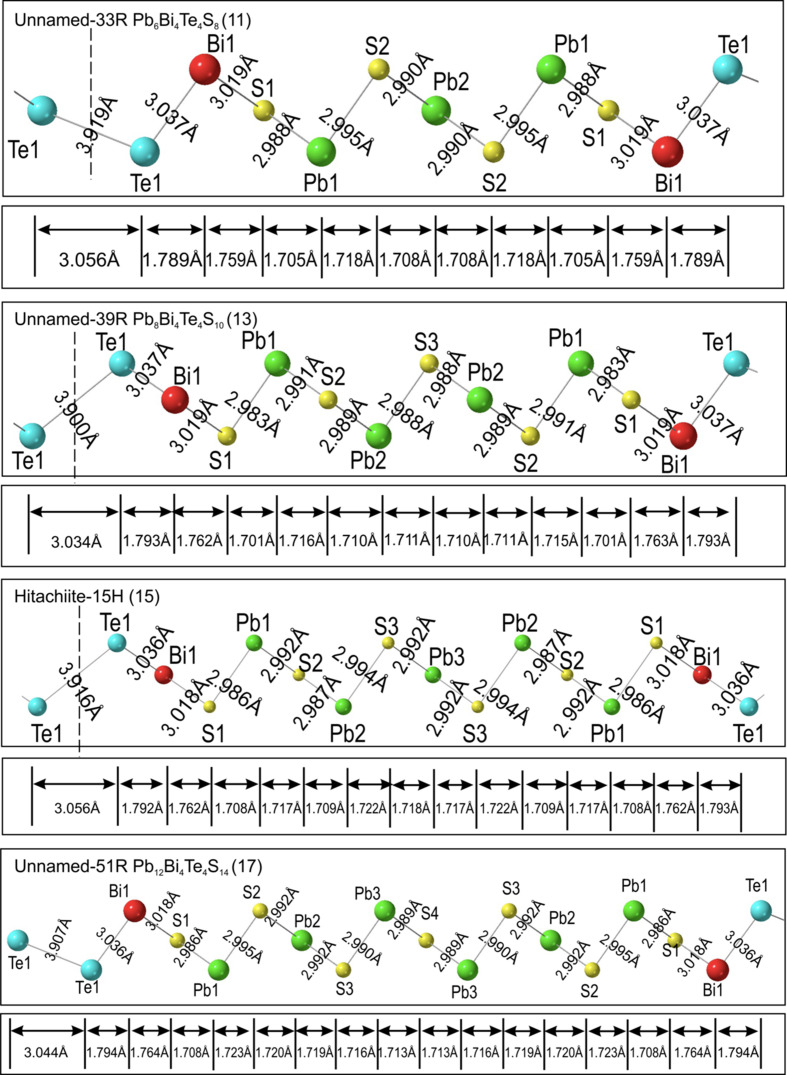
Bond types and lengths for the relaxed structures of phases where *n* = 6, 8, 10 and 12 within the asymmetric unit cell plotted on 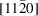 zone axis. Red = Bi, blue = Te, yellow = sulfur, green = Pb. Projections of bonds along the *c* axis are labelled on the bottom. They represent the bond length contributions to *d*_sub_ shown in Fig. 8 ▸.

**Table 1:** Background and crystal structure information for studied phases from the aleksite series

Mineral name	Explicit formula	*n*	Module stacks	*N*_1_	Polytype notation	Space group	*N* total	*c* calc (Å)	Reference	*c* (Å)	*a* (Å)	*d*_sub_ (Å)
Tetradymite	Bi_2_Te_2_S	0	5	5	15R	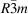	15	30	Pauling (1975 ▸)	29.589	4.238	1.973
Aleksite	Pb_2_Bi_4_Te_4_S_4_	2	7	7	21R	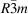	21	42	Liu & Chang (1994 ▸)	39.830	4.230	1.897
			5.9	14	42R		42	84	Lipovetskiy *et al.* (1979 ▸)	79.760	4.238	1.899
									Cook *et al.* (2007*a* ▸)	79.640	4.240	1.896
Saddlebackite	Pb_4_Bi_4_Te_4_S_6_	4	9	9	9H	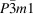	9	18	Liu & Chang (1994 ▸)	16.710	4.230	1.857
			7.11	18	18H		18	36	Clarke (1997 ▸)	33.400	4.230	1.856
Unnamed	Pb_6_Bi_4_Te_4_S_8_	6	11	11	33R	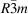	33	66	Ciobanu *et al.* (2009 ▸)	66.000	4.230	2.000
Unnamed	Pb_8_Bi_4_Te_4_S_10_	8	13	13	39R	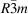	39	76		76.000	4.230	2.000
Hitachiite	Pb_10_Bi_4_Te_4_S_12_	10	15	15	15H	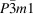	15	30	Kuribayashi *et al.* (2019 ▸)	27.02	4.22	1.801
Unnamed	Pb_12_Bi_4_Te_4_S_14_	12	17	17	51R	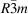	17	51	Ciobanu *et al.* (2009 ▸)	102.000	4.230	2.000

**Table 2:** Number of atoms and chemical formula units and *k* points employed in each simulation box for all nine aleksite series structures

	Bi_2_Te_2_S	PbBi_2_Te_2_S_2_	PbBi_2_Te_2_S_2_	Pb_2_Bi_2_Te_2_S_3_	Pb_2_Bi_2_Te_2_S_3_	Pb_3_Bi_2_Te_2_S_4_	Pb_4_Bi_2_Te_2_S_5_	Pb_5_Bi_2_Te_2_S_6_	Pb_6_Bi_2_Te_2_S_7_
Module stacks	5	7	5.9	9	7.11	11	13	15	17
Number of atoms	15	21	42	9	18	33	39	15	51
Formula units	3	3	6	1	2	3	3	1	3
*k*-point mesh	14 × 14 × 2	18 × 18 × 2	19 × 19 × 1	16 × 16 × 4	16 × 16 × 2	15 × 15 × 1	18 × 18 × 1	18 × 18 × 3	24 × 24 × 1

**Table 3:** Fitted equation of state parameters for nine minerals and unnamed phases from the aleksite series *E*_0_ is the reference energy, *V*_0_ is the equilibrium volume per atom for each simulated cell, *K*_0_ and *K*_0_′ are the bulk modulus and its derivative. *V*_0_ is compared with available experimental data from the literature.

Chemical formula	*E*_0_ (eV)	*V*_0_ (Å^3^)	*K*_0_ (GPa)	*K*_0_′	Ref.
Bi_2_Te_2_S	−4.18	31.83	25	8.3	This work
		30.68			[1]
Pb_2_Bi_4_Te_4_S_4_ (7)	−4.30	30.39	30	7.3	This work
		29.39			[2]
Pb_2_Bi_4_Te_4_S_4_ (5.9)	−4.30	30.38	30	7.6	This work
		29.54; 29.52			[3]; [4]
Pb_4_Bi_4_Te_4_S_6_ (9)	−4.37	29.56	33	6.8	This work
		28.77, 28.75			[5]
Pb_4_Bi_4_Te_4_S_6_ (7.11)	−4.37	29.53	34	6.7	This work
Pb_6_Bi_4_Te_4_S_8_	−4.41	29.03	36	6.5	This work
Pb_8_Bi_4_Te_4_S_10_	−4.43	28.59	41	5.6	This work
Pb_10_Bi_4_Te_4_S_12_	−4.45	28.39	39	5.9	This work
		27.78			[6]
Pb_12_Bi_4_Te_4_S_14_	−4.47	28.19	40	5.9	This work

[1] Pauling (1975 ▸). [2] Liu & Chang (1994 ▸). [3] Lipovetskiy *et al.* (1979 ▸). [4] Cook *et al.* (2007*a* ▸). [5] Clarke (1997 ▸). [6] Kuribayashi *et al.* (2019 ▸).

**Table 4:** Calculated unit-cell parameters *a* and *c* for nine relaxed structures based on density functional theory Values for the interlayer distance *d*_sub_ are obtained from the *c* unit-cell parameter and total number of layers (*N* total) for each phase.

Name	Formula	*n*	Space group	*a* (Å)	*c* (Å)	Volume (Å^3^)	*Z*	Density (g cm^−3)^	*d*_sub_ (Å)
Tetradymite-15R (5)	Bi_2_Te_2_S	0	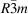	4.285	30.023	477.499	3	7.3579	2.002
Aleksite-21R (7)	Pb_2_Bi_4_Te_4_S_4_	2	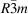	4.270	40.413	638.156	3	7.3733	1.924
Aleksite-42R (5.9)	Pb_2_Bi_4_Te_4_S_4_	2	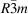	4.272	80.730	1276.062	6	7.3734	1.922
Saddlebackite-9H (9)	Pb_4_Bi_4_Te_4_S_6_	4	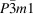	4.258	16.943	266.017	1	7.3895	1.883
Saddlebackite-18H (7.11)	Pb_4_Bi_4_Te_4_S_6_	4	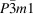	4.261	33.818	531.622	2	7.3952	1.879
Unnamed-33R (11)	Pb_6_Bi_4_Te_4_S_8_	6	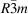	4.250	61.242	958.117	3	7.3990	1.856
Unnamed-39R (13)	Pb_8_Bi_4_Te_4_S_10_	8	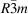	4.245	71.459	1115.068	3	7.4265	1.832
Hitachiite-15H (15)	Pb_10_Bi_4_Te_4_S_12_	10	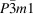	4.243	27.311	425.775	2	7.4162	1.821
Pb_12_Bi_4_Te_4_S_14_-51R (17)	Pb_12_Bi_4_Te_4_S_14_	12	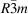	4.242	92.276	1437.842	3	7.4173	1.809

**Table 5:** DFT calculated formation energy (*E*_f_) and energy of layer mixing (*E*_mixing_) for the nine phases from the aleksite series Corresponding equations (2) and (3) are given in the text.

Formula	*E*_f_ (meV per atom)	*E*_mixing_ (meV per atom)
Bi_2_Te_2_S	−293.60	0
Pb_2_Bi_4_Te_4_S_4_ (7)	−377.05	−5.30
Pb_2_Bi_4_Te_4_S_4_ (5.9)	−377.05	−5.30
Pb_4_Bi_4_Te_4_S_6_ (9)	−419.11	−3.94
Pb_4_Bi_4_Te_4_S_6_ (7.11)	−419.11	−3.94
Pb_6_Bi_4_Te_4_S_8_	−446.03	−3.23
Pb_8_Bi_4_Te_4_S_10_	−464.36	−2.44
Pb_10_Bi_4_Te_4_S_12_	−478.07	−2.12
Pb_12_Bi_4_Te_4_S_14_	−488.73	−2.05

**Table 6:** Fitted equation of state parameters for four elements (Pb, S, Bi, Te) and endmember PbS *E*_0_ represents the reference energy, *V*_0_ is the equilibrium volume per atom for each simulated cell, *K*_0_ and *K*_0_′ are the bulk modulus and its derivative, respectively. *V*_0_ and *K*_0_ are compared with the available experimental data.

	*E*_0_/atom (eV)	*V*_0_ (Å^3^)	*K*_0_ (GPa)	*K*_0_′	Reference
Pb	−3.80	30.88	43	4.8	This study
		30.33	46		[1]; [2]
S	−4.24	27.12	8	6.2	This study
		25.76	8		[3]; [2]
Bi	−4.19	35.49	36	5.7	Yao *et al.* ▸(in the press)
		35.07	31		[4]; [2]
Te	−3.41	33.30	29	5.8	Yao *et al.* ▸(in the press)
		33.94	64		[5]; [2]
PbS	−4.59	26.61	54	3.8	This study
		26.09	48–73		[6]; [7,8]

[1] Wyckoff (1963 ▸). [2] https://periodictable.com/Properties/A/BulkModulus.html. [3] Rettig & Trotter (1987 ▸). [4] Schiferl & Barrett (1969 ▸). [5] Adenis *et al.* (1989 ▸). [6] Noda *et al.* (1987 ▸). [7] Padaki *et al.* (1981 ▸). [8] Littlewood (1980 ▸).

## References

[bb1] Adenis, C., Langer, V. & Lindqvist, O. (1989). *Acta Cryst.* C**45**, 941–942.

[bb2] Amelinckx, S., Van Tendeloo, G., Van Dyck, D. & Van Landuyt, J. (1989). *Phase Transit.* **16**, 3–40.

[bb3] Belmonte, D., Ottonello, G. & Zuccolini, M. V. (2014). *Am. Mineral.* **99**, 1449–1461.

[bb4] Blöchl, P. E. (1994). *Phys. Rev. B*, **50**, 17953–17979. 10.1103/physrevb.50.17953 9976227

[bb5] Ciobanu, C. L., Pring, A., Cook, N. J., Self, P., Jefferson, D., Dima, G. I. & Melnikov, V. (2009). *Am. Mineral.* **94**, 517–534.

[bb6] Clarke, R. M. (1997). *Aust. J. Mineral.* **3**, 119–124.

[bb7] Cook, N. J., Ciobanu, C. L., Liu, W., Slattery, A., Wade, B. P., Mills, S. J. & Stanley, C. J. (2019). *Minerals*, **9**, 628.

[bb8] Cook, N. J., Ciobanu, C. L., Slattery, A., Wade, B. P., Ehrig, K. (2021). *Proc. 3rd Eur. Mineral. Conf.* 29 August–2 September 2021, Krakow, Poland. Abstracts, p. 100.

[bb9] Cook, N. J., Ciobanu, C. L., Stanley, C. J., Paar, W. H. & Sundblad, K. (2007*a*). *Can. Mineral.* **45**, 417–435.

[bb10] Cook, N. J., Ciobanu, C. L., Wagner, T. & Stanley, C. J. (2007*b*). *Can. Mineral.* **45**, 665–708.

[bb11] Frangis, N., Kuypers, S., Manolikas, C., Van Tendeloo, G., Van Landuyt, J. & Amelinckx, S. (1990). *J. Solid State Chem.* **84**, 314–334.

[bb12] Grimme, S., Antony, J., Ehrlich, S. & Krieg, H. (2010). *J. Chem. Phys.* **132**, 154104. 10.1063/1.3382344 20423165

[bb13] Hohenberg, P. & Kohn, W. (1964). *Phys. Rev.* **136**, B864–B871.

[bb14] Ishizuka, K. (2002). *Ultramicroscopy*, **90**, 71–83. 10.1016/s0304-3991(01)00145-0 11942640

[bb100] Kresse, G. & Furthmüller, J. (1996). *Phys. Rev.* **54**, B11169. 10.1103/physrevb.54.11169 9984901

[bb15] Kohn, W. & Sham, L. J. (1965). *Phys. Rev.* **140**, A1133–A1138.

[bb16] Kuribayashi, T., Nagase, T., Nozaki, T., Ishibashi, J., Shimada, K., Shimizu, M. & Momma, K. (2019). *Mineral. Mag.* **83**, 733–739.

[bb17] Lind, H. & Lidin, S. (2003). *Solid State Sci.* **5**, 47–57.

[bb18] Lipovetskiy, A. G., Borodayev, Y. S. & Zav’yaiov, Y. N. (1979). *Int. Geol. Rev.* **21**, 1223–1228.

[bb19] Littlewood, P. B. (1980). *J. Phys. C. Solid State Phys.* **13**, 4855–4873.

[bb20] Liu, H. & Chang, L. L. Y. (1994). *Am. Mineral.* **79**, 1159–1166.

[bb21] Moëlo, Y., Makovicky, E., Mozgova, N. N., Jambor, J. L., Cook, N., Pring, A., Paar, W., Nickel, E. H., Graeser, S., Karup-Møller, S., Balic-Žunic, T., Mumme, W. G., Vurro, F. & Topa, D. (2008). *Eur. J. Mineral.* **20**, 7–62.

[bb22] Murnaghan, F. D. (1944). *Proc. Natl Acad. Sci. USA*, **30**, 244–247. 10.1073/pnas.30.9.244 PMC1078704 16588651

[bb23] Noda, Y., Masumoto, K., Ohba, S., Saito, Y., Toriumi, K., Iwata, Y. & Shibuya, I. (1987). *Acta Cryst.* C**43**, 1443–1445.

[bb24] Padaki, V. C., Lakshmikumar, S. T., Subramanyam, S. V. & Gopal, E. S. R. (1981). *Pramana J. Phys.* **17**, 25–32.

[bb25] Park, S., Ryu, B. & Park, S. (2021). *Appl. Sci.* **11**, 11341.

[bb26] Pauling, L. (1975). *Am. Mineral.* **60**, 994–997.

[bb27] Pennycook, S. J. & Jesson, D. E. (1990). *Phys. Rev. Lett.* **64**, 938–941. 10.1103/PhysRevLett.64.938 10042119

[bb28] Perdew, J. P., Ruzsinszky, A., Csonka, G. I., Vydrov, O. A., Scuseria, G. E., Constantin, L. A., Zhou, X. & Burke, K. (2008). *Phys. Rev. Lett.* **100**, 136406. 10.1103/PhysRevLett.100.136406 18517979

[bb29] Rettig, S. J. & Trotter, J. (1987). *Acta Cryst.* C**43**, 2260–2262.

[bb30] Schiferl, D. & Barrett, C. S. (1969). *J. Appl. Cryst.* **2**, 30–36.

[bb32] Shelimova, L. E., Karpinskii, O. G., Kosyakov, V. I., Shestakov, V. A., Zemskov, V. S. & Kuznetsov, F. A. (2000). *J. Struct. Chem.* **41**, 81–87.

[bb31] Shelimova, L. E., Karpinskii, O. G., Svechnikova, T. E., Avilov, E. S., Kretova, M. A. & Zemskov, V. S. (2004). *Inorg. Mater.* **40**, 1264–1270.

[bb33] Spiridonov, E. M. (1995). *Zap. Vses. Mineral. Ova*, **124**, 24–39.

[bb34] Woodcox, M., Young, J. & Smeu, M. (2019). *Phys. Rev. B*, **100**, 104105.

[bb35] Wyckoff, R. W. (1963). *Crystal Structures*, Vol. 1, p. 312. New York: Interscience.

[bb36] Yao, J., Ciobanu, C. L., Cook, N. J., Ehrig, K., Dima, G., Steinle-Neumann, G. (2023). *Am. Mineral.* https://doi.org/10.2138/am-2023-9018.

